# Vitamin D Deficiency: Insights and Perspectives from a Five-Year Retrospective Analysis of Children from Northeastern Romania

**DOI:** 10.3390/nu16223808

**Published:** 2024-11-07

**Authors:** Gabriela Ghiga, Elena Țarcă, Viorel Țarcă, Elena Lia Spoială, Gabriela Păduraru, Nicoleta Gimiga, Laura Otilia Boca, Otilia Iftinchi, Mădălina Andreea Donos, Lorena Mihaela Manole, Laura Mihaela Trandafir

**Affiliations:** 1 Department of Mother and Child, Faculty of Medicine, University of Medicine and Pharmacy “Grigore T. Popa”, 700115 Iasi, Romania; gabriela.ghiga@umfiasi.ro (G.G.); paduraru.gabriela@umfiasi.ro (G.P.); nicoleta.chiticariu@umfiasi.ro (N.G.); laura.boca@umfiasi.ro (L.O.B.); otilia.iftinchi@umfiasi.ro (O.I.); madalina.donos@umfiasi.ro (M.A.D.); lorena.manole@umfiasi.ro (L.M.M.); laura.trandafir@umfiasi.ro (L.M.T.); 2 “Sfânta Maria” Emergency Hospital for Children, 700309 Iasi, Romania; tarca.elena@umfiasi.ro; 3 Department of Pediatric Surgery, Faculty of Medicine, University of Medicine and Pharmacy “Grigore T. Popa”, 700115 Iasi, Romania; 4 Faculty of Dental Medicine, Apollonia University, 700511 Iași, Romania; vtarca@gmail.com; 5 Department of Concrete Structures, Building Materials, Technology and Management, Faculty of Civil Engineering and Building Services, “Gheorghe Asachi” Technical University, 700050 Iaşi, Romania

**Keywords:** vitamin D deficiency, children, nutritional status, obesity, underweight

## Abstract

Background: Vitamin D plays an important role in maintaining bone health with numerous benefits for extraskeletal health as well. Objectives: We aimed to assess the prevalence of vitamin D deficiency in children (0–18 years old) in a tertiary hospital in Romania between August 2019 and January 2024 and to investigate the role of adequate supplementation in this vulnerable population. Methods: The serum 25(OH)D levels were measured using a chemiluminescence binding assay. Results: A total of 744 participants were included in this study: 396 female (53.23%) and 348 male (46.77%). The serum levels of 25(OH)D ranged between 2.2 and 125.4 ng/mL, with a mean value of 27.4 ng/mL and a median value of 23.5 ng/mL. According to the cutoff values for the definition of vitamin D status (severe deficiency: <10 ng/mL, deficiency: <20 ng/mL, insufficiency: 20–29 ng/mL, and sufficiency: ≥30 ng/mL), the sample consisted of 77 (10.34%) cases of severe deficiency, 221 (29.7%) cases of deficiency, 194 (26.07%) cases of insufficiency, and 245 (32.93%) cases of sufficiency. There were seven cases of hypervitaminosis D with values above 100 ng/mL. The mean values (and corresponding 95% confidence intervals, CIs) were as follows: 27.85 ng/mL [25.95–29.76] in the males, 22.45 ng/mL [25.12–28.82] in the females, 24.82 ng/mL [21.86–27.77] in the spring, 28.62 ng/mL [26.42–30.81] in the summer, 32.30 ng/mL [29.16–35.44] in the autumn, and 24.01 ng/mL [21.57–26.44] in the winter. We observed a notable decline in the serum 25(OH)D levels with age, with 82.08% of the children in the age group above 6 years old having serum 25(OH)D levels below 30 ng/mL. In obese subjects, a higher prevalence of hypovitaminosis D was observed compared to non-obese subjects, with a mean value of 19.54 [17.50–21.57] ng/mL in obese children versus 28.89 [27.39–30.40] ng/mL in normal weight children. Conclusions: In our sample, the mean serum concentration of 25(OH)D was 27.4 ng/mL. Notably, 66.11% of the cases demonstrated varying degrees of hypovitaminosis D, with a significantly higher prevalence of 86.16% observed in the obese group. Healthcare providers should prioritize routine screening for vitamin D levels in pediatric patients with obesity to facilitate timely intervention and personalized supplementation strategies tailored to individual needs

## 1. Introduction

The history of vitamin D spans over 350 years, beginning in the early 1600s with the first documented cases of rickets [1]. While initial descriptions lacked precise medical distinctions from other bone diseases, historical treatises and lithographs from that era strongly suggested a link to vitamin D deficiency. However, it was not until the early 1900s to 1920s that the cause of vitamin D deficiency was elucidated, being attributed to sunlight exposure, and the chemical structures of vitamins D2 and D3 were identified [2]. Subsequent advancements led to the discovery of the active form of vitamin D, 1,25-dihydroxyvitamin D (1,25-(OH)2D), in 1967, with the unveiling of its metabolic pathways [3]. Further research revealed the complex mechanisms dedicated to vitamin D, including the transport proteins, metabolic enzymes, and vitamin D receptor (VDR), facilitating its diverse physiological effects beyond bone health in various tissues throughout the body [4].

Nowadays, vitamin D is recognized as a steroidal compound essential for all vertebrates including humans that plays a crucial role in regulating the blood levels of calcium and phosphate within a tightly controlled range. This function is vital for the maintenance of skeletal integrity, muscle contractility, immune response, and optimal cellular processes [5]. The threshold value for hypovitaminosis D is a subject of ongoing debate [6]. However, adequate levels are considered as those exceeding 30 ng/mL (75 nmol/L) [6]. Nutritional status significantly influences vitamin D levels, with both obesity and underweight conditions presenting unique challenges. In individuals with obesity, excess adipose tissue can sequester vitamin D, leading to lower bioavailability and increased risk of deficiency despite adequate dietary intake [7]. Conversely, underweight individuals may have insufficient dietary sources of vitamin D, which can also result in deficiency [8]. The adequate identification and treatment of this deficiency are paramount in mitigating adverse health outcomes and improving overall well-being in this vulnerable population.

In Romania, the data regarding vitamin D status, particularly in the northeastern region, are notably limited. In 2015, Chirita-Emandi et al. conducted a nationwide study of vitamin D levels in individuals aged 0 to 85 years, finding that approximately 59% had suboptimal levels. The prevalence of deficiency was particularly concerning, with 22% classified as deficient and 33% as insufficient [9]. More recently, Badiu et al. found that 27% of pediatric patients aged 2 to 18 years admitted to a university pediatric hospital in the northwestern region had vitamin D deficiency [10]. In this context, the objective of our study was to evaluate the prevalence of 25-hydroxyvitamin D deficiency in children aged 0 to 18 years at a tertiary hospital in Romania, as well as to examine the relationship between vitamin D levels and nutritional status.

## 2. Materials and Methods

We performed a retrospective study on children evaluated in “Sfânta Maria” Emergency Hospital for Children Iasi, Romania, between August 2019 and January 2024. We conducted a thorough examination of the participants’ medical documentation to gather information on their height, weight, season of presentation, and serum concentrations of 25-hydroxyvitamin D (25(OH)D).

Their obesity status was defined as having a Body Mass Index (BMI) greater than or equal to the 95th percentile for their age and gender in children aged two years and older or as a weight for height above the 95th percentile for children less than 2 years old. Underweight was defined as a weight for age of <5th percentile.

The children were evaluated during routine check-ups. We excluded those with conditions that could potentially impact vitamin D metabolism, such as the following:

Acute conditions: infections (including respiratory infections, gastroenteritis, urinary tract infections, and COVID-19), trauma, surgery, and burns.

Chronic diseases: chronic kidney disease, proximal tubulopathies, chronic liver diseases, neoplasms, autoimmune diseases, and allergic reactions.

Furthermore, we excluded all children receiving medications that could potentially influence vitamin D levels, including the following: antiepileptic medications (such as phenytoin, carbamazepine, and phenobarbital), antacids, diuretics, and corticosteroids. The serum 25(OH)D levels were measured using a chemiluminescence binding assay. The values are reported in ng/mL; to convert 25(OH)D to nmol/L, we multiplied by 2.496. According to the cutoff values for the definition of their serum 25(OH)D status, the patients were divided into four groups: the severe deficiency (25(OH)D < 10 ng/mL), deficiency (25(OH)D < 20 ng/mL), insufficiency (25(OH)D 20–29 ng/mL), and sufficiency (25(OH)D ≥ 30 ng/mL) groups. A vitamin D toxicity level was considered to be above 100 ng/mL; these cases were analyzed separately.

The data obtained were analyzed using the specific statistical functions provided by the IBM SPSS software version 25, such as the Shapiro–Wilk test and the chi-squared test. The descriptive statistics for the quantitative data were presented as the means and medians. The qualitative data were expressed as numbers (*n*) and percentages (%). The *p* values < 0.05 were considered statistically significant.

The study protocol was reviewed and approved by the Ethics Committee of “Sfânta Maria” Clinical Emergency Hospital for Children, Iasi (no. 24828/24 July 2024).

## 3. Results

A total of 744 participants were included in this study: 396 female (53.23%) and 348 male (46.77%). The serum levels of 25(OH)D ranged between 2.2 and 125.4 ng/mL, with a mean value of 27.4 ng/mL and a median value of 23.5 ng/mL. The mean values and corresponding 95% confidence intervals (CIs) were as follows: for the males, 27.85 ng/mL [25.95–29.76]; for the females, 22.45 ng/mL [25.12–28.82]; in the spring, 24.82 ng/mL [21.86–27.77]; in the summer, 28.62 ng/mL [26.42–30.81]; in the autumn, 32.30 ng/mL [29.16–35.44]; and in the winter, 24.01 ng/mL [21.57–26.44]. Additionally, the mean values and CIs for each age group were as follows: <1 year, 44.73 [39.96–49.50]; 1–3 years, 38.59 [34.92–42.26]; 3–6 years, 25.39 [22.92–27.86]; 6–12 years, 20.91 [19.43–22.39]; and 12–18 years, 18.34 [17.02–19.67].

According to the cutoff values for the definition of vitamin D status, the sample consisted of the following: 77 (10.34%) cases of severe deficiency, 221 (29.7%) cases of deficiency, 194 (26.07%) cases of insufficiency, and 245 (32.93%) cases of sufficiency. There were seven cases of hypervitaminosis D with values above 100 ng/mL. Table 1 illustrates the vitamin D levels in our sample (mean values by sex, age, season, and weight.

The distribution of vitamin D levels (severe deficiency, deficiency, insufficiency, and sufficiency) grouped by gender, age, season, and nutritional status are detailed in Table 2.

**Figure 1:**
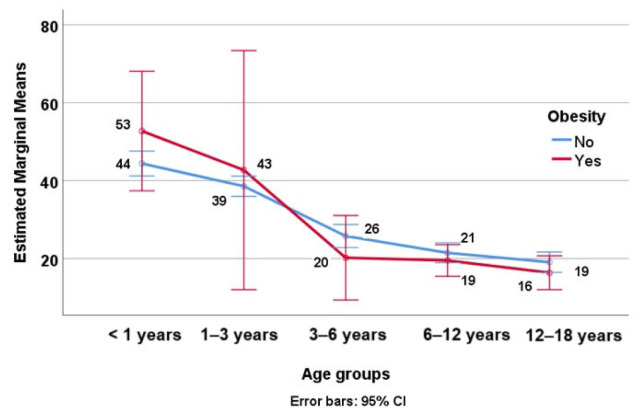
Average vitamin D levels by age group and obesity status.

In the obese subjects, we noticed a higher prevalence of hypovitaminosis D compared to the non-obese subjects: 13.33% of severe deficiency cases in the obesity group compared to 9.88% in the non-obesity group and 45.33% of deficiency cases in the obesity group compared to 26.9% in the non-obesity group. The values of vitamin D (mean ± SD) in both the obese and non-obese subjects are detailed in Table 3. Figure 1 illustrates the average vitamin D levels categorized by age group and obesity status, emphasizing the impact of obesity on vitamin D concentrations, especially in children > 3 years old.

**Figure 2:**
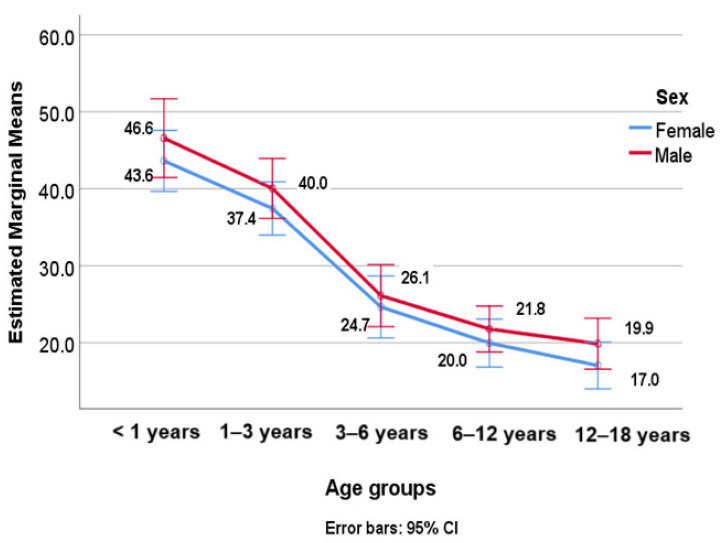
Average vitamin D levels by age group and sex.

A two-way ANOVA was conducted to examine the effect of age groups (Factor A: <1 year, 1–3 years, 3–6 years, 6–12 years, and 12–18 years) and sex (Factor B: male and female) on the vitamin D levels as the dependent variable (Figure 2). The results showed a significant main effect of the age groups—F(4, 734) = 71.758, *p* < 0.01, and η^2^ = 0.281 (moderate effect)—indicating that the younger patients, especially those under 3 years of age, had higher levels of vitamin D compared to the older patients. There was no significant main effect of sex—F(1, 734) = 3.746 and *p* = 0.053—and, also, no significant interaction effect—F(4, 734) = 0.058 and *p* = 0.994 (Age groups * Sex). The post hoc analysis revealed that the vitamin D levels differed significantly between the patients under 1 year of age or between 1–3 years of age and all other age groups (*p* < 0.05). There were no statistically significant differences between the patients aged 3–6 years and 6–12 years (*p* = 0.133) or between those aged 6–12 and 12–18 years (*p* = 1.000).

**Figure 3:**
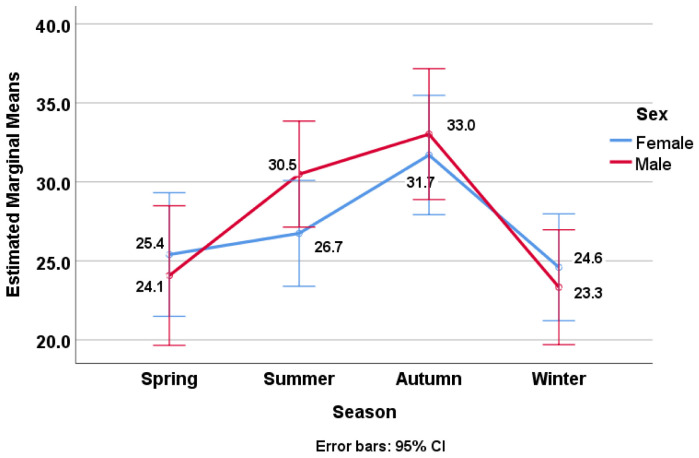
Average vitamin D levels by season and sex.

Secondly, a two-way ANOVA was conducted to examine the effect of seasons (Factor A: spring, summer, autumn, and winter) and sex (Factor B: male and female) on vitamin D levels as the dependent variable (Figure 3). The results showed a significant main effect of season—F(3, 736) = 7.869, *p* < 0.01, and η^2^ = 0.031—indicating that, in the summer and autumn, the patients had higher levels of vitamin D compared to in the spring and winter. Similar to the previous case, there was no significant main effect of sex—F(1, 736) = 0.209 and *p* = 0.648—and, also, no significant interaction effect—F(3, 736) = 0.898 and *p* = 0.442 (Seasons * Sex). The post hoc analysis revealed that the vitamin D levels differed significantly between the patients analyzed in the spring and the autumn, the summer and the winter, and also the autumn and the winter (*p* < 0.05). There were no statistically significant differences between Spring–Summer (*p* = 0.267), Spring–Winter (*p* = 1.000), and Summer–Autumn (*p* = 0.274).

**Figure 4:**
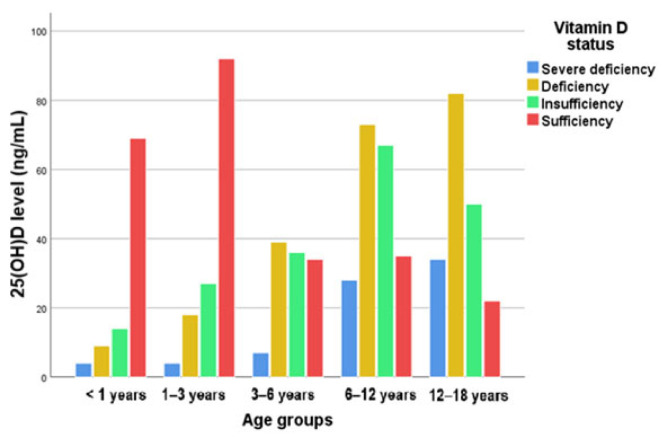
Vitamin D status according to age group.

The distribution of serum 25(OH)D levels grouped by age (<1 year old, 1–3 years old, 3–6 years old, 6–12 years old, and 12–18 years old) can be visualized in Figure 4.

In order to draw conclusions about the specific differences between categories and to assess which cells in the contingency table contributed the most to the previously calculated χ^2^ test value, we determined the standardized residuals for each pair of categories (Table 4). To assess the potential association between the age groups and vitamin D levels, we employed the chi-squared (χ^2^) test. The analysis yielded a chi-squared value of χ^2^(12) = 207.912, with a *p*-value less than 0.01. This result indicates a statistically significant relationship between the age groups and vitamin D levels, with the *p*-value suggesting that the likelihood of this association occurring by chance is less than 1%. Standardized residuals are important when interpreting the chi-squared test, as they help identify where significant differences between the observed and expected values exist in the contingency table. They show how much the observed values deviate from the expected values for each cell, relative to the variation expected under the null hypothesis. By examining the size and sign of these residuals, it is possible to identify which specific age groups deviate significantly from the expected vitamin D levels, providing more insight into the pattern of association between the age and vitamin D status.

Large positive residuals suggest higher than expected levels of vitamin D in certain age groups (the vitamin D status “Sufficiency” values for age groups “<1 years” and “1–3 years” are approximatively double the expected). The explanation is similar for groups “6–12 years” and “12–18 years” in the case of vitamin D statuses “Deficiency” and “Insufficiency” and “12–18 years” for “Severe Deficiency”.

Large negative residuals suggest lower than expected levels of vitamin D (the vitamin D status “Severe Deficiency”, “Deficiency”, and “Insufficiency” values for age groups “<1 years” and “1–3 years” are smaller than expected). The explanation is similar for groups “6–12 years” and “12–18 years” in the case of vitamin D status “Sufficiency”.

For “3–6 years”, all the values suggest that the distribution of vitamin D status aligns closely with what would be expected by chance.

We observed very high standardized values (over 1.96, for a significance level of 0.05—in green), which indicate significant deviations of the expected frequencies from the observed frequencies for the respective combinations of categories. For example, among patients under 1 year old with a sufficient vitamin D level, there are 69 patients, whereas we would have expected only 32 if there were no association between the variables.

## 4. Discussion

Vitamin D deficiency and insufficiency are widespread concerns globally [11]. In a large study of 7,947,359 participants from 81 countries, 15.7%, 47.9%, and 76.6% had serum 25-hydroxyvitamin D levels below 30, 50, and 75 nmol/L, respectively, with a slight decrease in prevalence from 2000–2010 to 2011–2022 [11]. Recent evaluations of global vitamin D levels indicate that North and Latin America, along with Australia, exhibit a superior vitamin D status compared to Europe [12]. Additionally, Southeastern Asia shows better vitamin D levels than India and Northern Asia, while Central Africa has a more favorable vitamin D status than both Northern and Southern Africa [12]. The Middle East generally reports the lowest vitamin D levels. Within Europe, Northern European countries demonstrate better vitamin D status than their southern and eastern counterparts [13]. A worldwide study on the prevalence and health impact of vitamin D deficiency revealed alarmingly high rates of severe deficiency among children, with figures reaching 61% in India, 86% in Iran, and 51% in Turkey [14]—it is noteworthy that our study found a significantly lower rate of deficiency at 10.34%. This contrast highlights the variability in vitamin D deficiency rates across different populations.

Although a serum 25(OH)D level below 10 ng/mL is recognized as the minimum threshold for vitamin D status [15], there have been reports of rickets occurring in infants and young children with a serum 25(OH)D concentration exceeding 10 ng/mL [16]. In 2011, the US Institute of Medicine (IOM) recommended a serum 25(OH)D level above 20 ng/mL for optimal bone health [17]. Subsequently, the Endocrine Society Task Force introduced a guideline defining vitamin D sufficiency as a serum 25(OH)D level greater than 30 ng/mL, insufficiency as 20–30 ng/mL, and deficiency as less than 20 ng/mL [18]. This classification was based on emerging evidence suggesting that the ideal 25(OH)D level for adults might be above 30 ng/mL [19].

It is important to note that, in Romania, particularly in the northeastern region, the data on vitamin D status remain significantly limited. This gap in knowledge emphasizes the need for further research to better understand and address vitamin D deficiency in children. Our findings reveal that 32.93% of individuals in the 0–18 age group exhibit sufficient serum 25(OH)D levels, contrasting with the results from a similar study from the northwestern region of Romania, which indicate a higher sufficiency rate of 40% [10]. Notably, our study shows a higher prevalence of deficiency (29.7% with 10.34% classified as severe) compared to Badiu et al.’s 27% deficiency rate [10]. Additionally, there are notable differences in serum 25(OH)D levels based on age, gender, and time of year.

Situated between 44° N and 48° N latitude in Eastern Europe, Romania experiences seasonal variations in vitamin D levels, reaching their highest points in September and their lowest in March [20]. In our research, most cases of severe deficiency occurred during spring, which is in accordance with the data reported from a pediatric hospital located in the northwestern region of Romania [10]. We also observed a notable decline in serum 25(OH)D levels with age among children aged 0–17 years old. Specifically, in the age group of children aged above 6 years old, 82.08% of individuals had serum 25(OH)D levels below 30 ng/mL, with this figure rising to 88.53% during adolescence. This trend is comparable with the results of Zhang et al., who reported in a Chinese sample that in the post-preschool age group, the proportion of individuals with serum 25(OH)D levels < 30 ng/mL exceeded 60%, reaching 90% in adolescence [21]. Furthermore, based on a prior investigation into vitamin D levels in American children, it was found that the prevalence of vitamin D deficiency and insufficiency rises with age. Specifically, in 2009, the rates were reported as 14%, 20%, and 28.8% for children aged 2–5 years, 6–11 years, and adolescents, respectively [22]. Our data are also consistent with the recent study of Van de Walle et al., which reported that the prevalence of vitamin D insufficiency along with vitamin D deficiency was highest in 7–12-year-old children and adolescents (13–18 years), being 24.9% and 20.7%, respectively [23]. In our study, a two-way ANOVA revealed a strong main effect of age, with younger patients, particularly those under 3 years, exhibiting higher vitamin D levels compared to older age groups. This finding aligns with Badiu et al., who reported that children aged 2–5 years had significantly higher median values than those aged 6–11 and 12–18 years [10]. Several factors may contribute to the decrease in vitamin D levels as individuals age. Firstly, the skin’s ability to synthesize vitamin D from sunlight diminishes with age, due to reduced epidermal thickness and changes in the skin’s composition [24]. Additionally, older children may spend less time outdoors, leading to decreased sun exposure, which is crucial for vitamin D production [25]. Dietary intake may also play a role, as adolescents often have different nutritional habits that may not meet the recommended levels of vitamin D [26]. In this context, the prophylaxis of vitamin D deficiency is crucial, especially as we age. Research indicates that the level of 7-dehydrocholesterol declines by over 50% between the ages of 20 and 80 [24].

While our data found no effect of sex on vitamin D levels, with a *p*-value of 0.053, indicating a trend but not reaching statistical significance, Badiu Tișa et al. identified a significant difference in serum 25(OH)D values between male and female patients, with the males exhibiting higher median values (*p* = 0.02) [10]. These contrasting results highlight the complexity of vitamin D status and suggest that further research is needed to understand how sex and age interact to influence vitamin D levels in different populations.

In obese subjects, we noticed a higher prevalence of hypovitaminosis D compared to non-obese subjects. This aligns with the findings from a Spanish study that reported an inverse correlation between BMI and serum vitamin D levels [27]. The obese and overweight participants exhibited a 6.29-fold and 8.56-fold increased risk of vitamin D deficiency, respectively, in a study of 383 participants aged between 10 and 19 years old [28]. Furthermore, a meta-analysis of 20 studies revealed that the relative risk for an association between obesity and vitamin D deficiency in the pediatric population was 1.41 (95% CI: 1.26–1.59) (I^2^ = 89%, *p* < 0.01) [29]. In this context, it is important to determine whether the relationship between hypovitaminosis D and obesity is based on coincidence or consequence [30]. Numerous theories have been proposed to account for the reduced levels of 25(OH)D observed in individuals with obesity. This relationship may be attributed to factors such as decreased skin exposure to sunlight, altered vitamin D metabolism, and the sequestration of vitamin D in adipose tissue [31]. However, the idea that the increased adipose tissue may serve as a larger reservoir of vitamin D was contested by Drincic et al., who suggested that, in individuals with obesity, the 25(OH)D levels are simply diluted due to a greater blood volume, supporting the volumetric dilution hypothesis [32]. Additionally, obesity-related secondary hyperparathyroidism could lead to the decreased hepatic production of 25(OH)D [33]. Other theories have emerged, indicating potential alterations in vitamin D metabolism, particularly within adipose tissue, where lower levels of CYP2J2 mRNA have been observed in obese women compared to their lean counterparts [34]. In light of the increasing prevalence of obesity and the fact that vitamin D is fat-soluble, there is a growing indication that individuals in this category may require higher doses of vitamin D, potentially ranging from 1000 to 1500 IU per day [35].

In underweight children, we observed that 43.47% had sufficient levels of vitamin D, in contrast to only 13.33% of children in the obesity group. This may be for several reasons: the adipose tissue sequestration of vitamin D; unhealthy dietary patterns; reduced sun exposure due to a sedentary lifestyle; and chronic low-grade inflammation, which can interfere with the metabolism of vitamin D and its bioavailability [36]. These factors combined can lead to a higher prevalence of vitamin D deficiency in children with obesity compared to their underweight counterparts. It is important for healthcare providers to monitor vitamin D levels in children, especially those with obesity, to ensure they receive appropriate guidance and supplementation if necessary. Research indicates that vitamin D plays a crucial role in modulating immune responses, regulating inflammatory processes, and maintaining musculoskeletal health [37]. Furthermore, addressing vitamin D insufficiency early on can enhance treatment efficacy, reduce disease progression, and potentially lower the risk of the complications associated with chronic illnesses [38].

Understanding the risk factors for vitamin D deficiency is crucial for clinicians to effectively assess and implement appropriate supplementation strategies. The key populations at risk include infants who are exclusively breastfed or consume less than 1000 mL of infant formula per day, children residing north or south of the 33° latitudes, and those in urban or polluted areas [39]. Additionally, individuals who are obese, have darker skin pigmentation, or cover their skin for cultural reasons, as well as those with gastrointestinal disorders or on medications that impair vitamin D absorption, should be monitored [39]. Children in institutional settings, hospitals, or schools with restricted outdoor activity are also at an increased risk. While measuring the 25(OH)D levels can confirm vitamin D deficiency, the high prevalence of insufficiency across various populations often justifies initiating supplementation without this prior testing [39].

In pediatric populations, adequate levels of vitamin D are crucial not only for bone health but also for supporting the immune function and reducing the risk of chronic diseases later in life [6]. However, large supplementation trials recruiting vitamin D-replete adults (with a serum 25OHD concentration >50 nmol/L) have demonstrated no effects on the incidence of cancer, cardiovascular events, or type 2 diabetes mellitus and no benefits in terms of bone density and the risk of falls, but the correction of severe vitamin D deficiency remains essential [40]. Given that the dietary sources of vitamin D are often insufficient and that limited sun exposure can hinder endogenous synthesis, particularly in regions with low sunlight, supplementation becomes a vital strategy [41]. The optimal prevention and treatment includes daily exposure to natural sunlight for 15 to 30 min during the peak hours from 10 a.m. to 3 p.m., though this may not be accessible to all individuals at risk [42]. For oral supplementation, both vitamin D3 (cholecalciferol) and vitamin D2 (ergocalciferol) are available, with vitamin D3 being approximately 87% more effective in increasing and maintaining serum vitamin D concentrations and resulting in significantly greater storage [43]. The American Academy of Pediatrics (AAP) and the Institute of Medicine (IOM) recommend a daily intake of 400 IU of vitamin D3 [44], while the Canadian Pediatric Society suggests doses up to 800 IU to prevent rickets [45]. However, higher doses may be necessary to obtain additional health benefits. The evidence indicates that for every 100 IU of vitamin D3 consumed, the serum levels can increase by 1 ng/mL over a period of 3 to 4 months [46]. For children with existing deficiencies, a standard dose of 400 IU is often insufficient; studies have shown that only a dosage of 1600 IU/day effectively elevates the 25(OH)D levels above 28 ng/mL after three months [47]. Experts in vitamin D research recommend administering approximately 1000 IU per 11 kg of body weight daily for healthy children to achieve the optimal 25(OH)D levels throughout the year [48].

Education on the significance of vitamin D in pediatric health and disease management is crucial for fostering awareness and adherence to recommended adequate interventions. By integrating evidence-based practices for the identification and treatment of vitamin D deficiency into clinical protocols, healthcare providers can enhance the quality of care, especially in children with malnutrition (both undernutrition and obesity). In conclusion, recognizing the pivotal role of vitamin D in the context of malnutrition underscores the necessity of proactive screening, targeted interventions, and ongoing monitoring to optimize health outcomes and quality of life in this vulnerable patient demographic.

Our study presents several limitations that warrant consideration. Firstly, the research was conducted in a single tertiary hospital in Romania, which may limit the generalizability of the findings to broader populations, particularly in different geographical or socio-economic contexts. Another limitation of our study is the violation of the assumption of normality. The ANOVA assumes that the residuals (errors) are normally distributed, and this assumption was not fully met based on the normality tests (Shapiro–Wilk). This could potentially impact the validity of the results, particularly with small sample sizes. However, this element has little impact because our sample is generally considered large. With 744 participants, this study has a good potential for generalizability and also provides robustness against assumption violations, particularly for tests like the ANOVA. Due to the retrospective nature of this study and its anonymous design, the individual data on vitamin D supplementation, dietary intake, and sun exposure levels were not obtainable.

## 5. Conclusions

In our sample, we observed a notable decline in serum 25(OH)D levels with age, with 82.08% of children in the age group above 6 years old having serum 25(OH)D levels below 30 ng/mL. In obese subjects, a higher prevalence of hypovitaminosis D was observed compared to non-obese subjects. Despite being identified over a century ago, vitamin D deficiency continues to pose a significant public health challenge. Healthcare providers should prioritize routine screening for vitamin D levels in pediatric patients, especially in those with malnutrition, both undernutrition and obesity, to facilitate timely intervention and personalized supplementation strategies tailored to individual needs. Emphasizing the importance of maintaining adequate vitamin D status through supplementation, dietary modifications, and sunlight exposure is essential in optimizing therapeutic outcomes and promoting long-term health. Collaborative efforts between healthcare professionals, caregivers, and patients are instrumental in ensuring the comprehensive management of vitamin D deficiency.

## Figures and Tables

**Table 1:** Mean (SD) serum 25-Hydroxyvitamin D (ng/mL) by sex, age, season, and weight.

Groups	All		Males				Females	
	*n*	Mean (ng/mL) (SD)	*n*	Mean (ng/mL) (SD)			*n*	Mean (ng/mL) (SD)
Age								
All ages (years old)	744 *	27.38 (18.39)	348	27.85 (18.03)			396	26.97 (18.70)
<1			96	44.73 (23.55)	36	46.58 (21.58)	60	43.62 (24.77)
1–3			141	38.59 (22.02)	62	40.04 (21.92)	79	37.45 (22.18)
3–6			116	25.39 (13.43)	58	26.12 (16.15)	58	24.66 (10.10)
6–12			203	20.91 (10.68)	106	21.79 (10.94)	97	19.96 (10.36)
12–18			188	18.34 (9.21)	86	19.88 (9.95)	102	17.05 (8.36)
Season								
Spring			148	24.82 (18.18)	65	24.07 (15.81)	83	25.40 (19.91)
Summer			226	28.62 (16.76)	113	30.49 (17.52)	113	26.74 (15.82)
Autumn			163	32.30 (20.28)	74	33.02 (21.89)	89	31.70 (18.93)
Winter			207	24.01 (17.79)	96	23.33 (15.15)	111	24.59 (19.84)
Weight								
Obese			120	19.54 (11.24)	65	21.64 (13.51)	55	17.06 (7.11)
Normal weight			624	28.89 (19.10)	283	29.28 (18.65)	341	28.57 (19.49)

* Seven cases of hypervitaminosis D with values above 100 ng/mL were excluded.

**Table 2:** Distribution of vitamin D levels grouped by gender, age, season, and nutritional status.

Characteristics		Severe Deficiency *n* = 77 (%)	Deficiency *n* = 221 (%)	Insufficiency *n* = 194 (%)	Sufficiency *n* = 245 (%)	*p*-Value *
Gender	Boy	35 (10.14%)	89 (25.79%)	102 (29.56%)	119 (34.49%)	0.082
	Girl	42 (10.71%)	132 (33.67%)	92 (23.46%)	126 (32.14%)	
Age (years old)	<1	4 (4.25%)	9 (9.57%)	14 (14.89%)	67 (71.27%)	<0.001
	1–3	4 (2.75%)	19 (13.10%)	30 (20.68%)	92 (63.44%)	
	3–6	7 (6.03%)	39 (33.62%)	38 (32.75%)	32 (27.58%)	
	6–12	27 (13.43%)	72 (35.82%)	66 (32.83%)	36 (17.91%)	
	12–18	35 (18.22%)	84 (43.75%)	51 (26.56%)	22 (11.45%)	
Season	Spring	20 (13.51%)	60 (40.54%)	27 (18.24%)	41 (27.7%)	<0.001
	Summer	22 (9.77%)	46 (20.44%)	70 (31.11%)	87 (38.66%)	
	Autumn	9 (5.66%)	34 (21.38%)	48 (30.18%)	68 (42.76%)	
	Winter	26 (12.68%)	81 (39.51%)	49 (23.9%)	49 (23.9%)	
Nutritional status	Obese	16 (13.33%)	55 (45.33%)	33 (27.5%)	16 (13.33%)	<0.001
	Underweight	19 (6.35%)	67 (22.4%)	83 (27.75%)	130 (43.47%)	<0.001

* *p*-value according to the chi-squared test.

**Table 3:** Analysis of vitamin D levels (mean ± SD) in obese and non-obese subjects by age groups.

Age Groups	Obesity	Mean	Std. Deviation	N
<1 year	No	44.383	23.5553	92
	Yes	52.725	25.4751	4
	Total	44.730	23.5541	96
1–3 years	No	38.559	22.0976	140
	Yes	42.700	-	1
	Total	38.589	22.0213	141
3–6 years	No	25.772	13.6512	108
	Yes	20.200	9.0850	8
	Total	25.388	13.4323	116
6–12 years	No	21.466	11.2017	146
	Yes	19.495	9.1567	57
	Total	20.912	10.6819	203
12–18 years	No	19.063	9.8218	138
	Yes	19.495	9.1567	57
	Total	18.344	9.2068	188
Total	No	28.894	19.1015	624
	Yes	19.536	11.2413	120
	Total	27.384	18.3858	744

**Table 4:** Standardized residuals according to the expected values for vitamin D status.

Age Groups		Severe Deficiency	Deficiency	Insufficiency	Sufficiency	Total
<1 year	Number of children	4	9	14	69	96
	Expected	9.935	28.516	25.032	32.516	96
	Standardized residuals	−2.131	−4.671	−2.748	8.431	
1–3 years	Number of children	4	18	27	92	141
	Expected	14.593	41.883	36.766	47.758	141
	Standardized residuals	−3.253	−4.889	−2.081	8.745	
3–6 years	Number of children	7	39	36	34	11
	Expected	12.005	34.457	30.247	39.290	116
	Standardized residuals	−1.661	1.005	1.324	−1.130	
6–12 years	Number of children	28	73	67	35	203
	Expected	21.009	60.300	52.933	68.758	203
	Standardized residuals	1.889	2.288	2.637	−5.871	
12–18 years	Number of children	34	82	50	22	188
	Expected	19.457	55.844	49.022	63.677	188
	Standardized residuals	4.028	4.829	0.188	−7.430	

## Data Availability

The data presented in this study are available on request from the corresponding author due to ethical reasons.
